# Melatonin Alleviates the Impairment of Muscle Bioenergetics and Protein Quality Control Systems in Leptin-Deficiency-Induced Obesity

**DOI:** 10.3390/antiox12111962

**Published:** 2023-11-03

**Authors:** Yaiza Potes, Andrea Díaz-Luis, Juan C. Bermejo-Millo, Zulema Pérez-Martínez, Beatriz de Luxán-Delgado, Adrian Rubio-González, Iván Menéndez-Valle, José Gutiérrez-Rodríguez, Juan J. Solano, Beatriz Caballero, Ignacio Vega-Naredo, Ana Coto-Montes

**Affiliations:** 1 Department of Morphology and Cell Biology, Faculty of Medicine, University of Oviedo, 33006 Oviedo, Spain; 2 Instituto de Investigación Sanitaria del Principado de Asturias (ISPA), 33011 Oviedo, Spain; 3 Institute of Neurosciences of the Principality of Asturias (INEUROPA), 33006 Oviedo, Spain; 4 Microbiology Service, Central University Hospital of Asturias, 33011 Oviedo, Spain; 5 Immunology Service, Central University Hospital of Asturias, 33011 Oviedo, Spain; 6 Geriatric Service, Monte Naranco Hospital, 33012 Oviedo, Spain

**Keywords:** skeletal muscle, leptin, melatonin, obesity, metabolism, mitochondria

## Abstract

Leptin is critically compromised in the major common forms of obesity. Skeletal muscle is the main effector tissue for energy modification that occurs as a result of the effect of endocrine axes, such as leptin signaling. Our study was carried out using skeletal muscle from a leptin-deficient animal model, in order to ascertain the importance of this hormone and to identify the major skeletal muscle mechanisms affected. We also examined the therapeutic role of melatonin against leptin-induced muscle wasting. Here, we report that leptin deficiency stimulates fatty acid β-oxidation, which results in mitochondrial uncoupling and the suppression of mitochondrial oxidative damage; however, it increases cytosolic oxidative damage. Thus, different nutrient-sensing pathways are disrupted, impairing proteostasis and promoting lipid anabolism, which induces myofiber degeneration and drives oxidative type I fiber conversion. Melatonin treatment plays a significant role in reducing cellular oxidative damage and regulating energy homeostasis and fuel utilization. Melatonin is able to improve both glucose and mitochondrial metabolism and partially restore proteostasis. Taken together, our study demonstrates melatonin to be a decisive mitochondrial function-fate regulator in skeletal muscle, with implications for resembling physiological energy requirements and targeting glycolytic type II fiber recovery.

## 1. Introduction

Obesity is a burgeoning epidemic worldwide that is associated with cardiovascular disease, type 2 diabetes, musculoskeletal disorders, and certain cancers. Leptin is an adipocyte-derived hormone that modulates neuroendocrine axes and metabolic cues. A decrease in leptin levels and leptin resistance are considered the main contributing factors to obesity by inducing a hyperphagic phenotype and nutrient overload. Skeletal muscle is at the crossroads of fatty acid and glucose metabolism, and thus it is a crucial parameter for decisive metabolic adaptations. Moreover, skeletal muscle accounts for 40% of the total body weight in lean individuals and represents 30–40% of the resting metabolic rate [1]. A direct effect of leptin on skeletal muscle may contribute to the development of obesity and insulin resistance. Indeed, previous investigations demonstrated that leptin directly increases muscle fat oxidation and glucose uptake, and enhances insulin signaling in skeletal muscle fibers [2,3]. However, the molecular mechanisms underlying skeletal muscle dysfunction as a result of leptin-signaling disruption remain unclear.

Accumulating evidence has shown that obesity can alter muscle stem cells by reducing their function and number and impairing differentiation and regeneration capability [4,5]. Many works reported that the altered myogenic differentiation and regeneration processes observed in obesity are attributed to dysfunctional mitochondria and reduced oxidative phosphorylation (OXPHOS) capacity [6]. Interestingly, transcriptome analyses revealed that leptin influences muscle regeneration [7], and its deficiency is associated with reduced regenerative potential [8], supporting the pivotal role of leptin in the regulation of muscle mass and function. Studies conducted in mice indicate that leptin is capable of acting within the brain to change energy expenditure and thermoregulatory circuits in skeletal muscle [9]. Leptin was found to modify mitochondrial function in order to regulate heat production, which is indicative of an adaptive thermogenic strategy [10]. Therefore, leptin-signaling disruption may have an impact on mitochondrial function, which may ultimately affect myogenic capacity and muscle mass deterioration. The close interaction between leptin and cellular bioenergetic metabolism has raised interest in the scientific community, but there are still many research gaps to explore. Leptin treatment was found to promote ATP production through mitochondrial respiration in cancer cells [11] and enhance mitochondrial fusion-dependent glycolysis in mesenchymal stem cells [12]. Current data also suggest that a nutrient supply–demand imbalance, such as that caused by leptin dysregulation, may affect muscle mitochondria function due to the increased toxic intermediates of fatty acid metabolism [13,14,15]. To date, little effort has been made to study the role of leptin in muscle mitochondria metabolism and its link with obesity. Therefore, the mitochondrial alterations underlying skeletal muscle dysfunction as a result of leptin-signaling disruption remain unclear. On the other hand, given the postmitotic nature of skeletal muscle cells, quality control mechanisms seem to be decisive for maintaining muscle homeostasis. In particular, autophagy facilitates the adaptation to nutritional stress. Autophagy, which is mainly regulated by the nutrient-sensing mTOR pathway, supports bioenergetic demands and contributes to metabolic adaptation during cell fate determination [16]. Over the past few years, some studies have revealed that leptin seems to impact the way autophagy works, but these studies are controversial. In one study, circulating levels of leptin were found to be positively associated with increased autophagic response in adipocytes [17], whereas in another study, it was shown to inhibit the autophagy of chondrocytes [18]. However, there is scarce evidence that can be used to develop a hypothesis regarding how leptin impacts the autophagic response of skeletal muscle tissue. Overall, previous research appears to show that both mitochondrial function and quality control mechanisms could be potential targets for developing obesity-related muscle atrophy. However, although previous studies have provided clues, there is no evidence about the role of leptin in muscle mitochondria metabolism and autophagic response and its association with obesity.

New research has demonstrated that the muscle circadian clock regulates normal metabolic homeostasis and may be involved in obesity development [19,20,21]. Recently, decreased melatonin peak was found in obese rodents [22]. Indeed, it has been described that melatonin drives the circadian rhythm of leptin, impacting the modulation of food intake and energy balance [23,24,25]. Melatonin is a widespread signaling molecule involved in the circadian rhythm, the radical scavenging system, cell death, and inflammation [26,27]. Melatonin was also found to restore adipokine patterns and metabolism in diet-induced obesity [28,29,30]. This neurohormone is also considered an important autophagy regulator and mitochondrial protector in obesity and other disorders [26]. It has gained attention driven by the finding that melatonin accumulates in mitochondria at high concentrations, supporting the existence of regulatory mechanisms in these organelles [31]. Although studies on melatonin have transformed obesity research, therapeutic applications of this molecule are still limited due to the complexity and poor understanding of the relationship between melatonin and leptin.

To determine the role of leptin in muscle bioenergetics and quality control, in this study, we examined the skeletal muscles of leptin-deficient obese mice. We hypothesized that the lack of leptin induces metabolic reprogramming in skeletal muscle, compromising mitochondrial function and autophagic response, which may be critical for muscle mass and functional performance. We also speculated that melatonin administration may overcome the effects of leptin deficiency. Melatonin could alleviate leptin deficiency by modulating muscle mitochondrial function and autophagic response.

## 2. Materials and Methods

### 2.1. Animals

Male wild-type (C57BL/6J) and leptin-deficient ob/ob (B6.VLepob/J) mice at six weeks of age were purchased from Charles River Laboratory (Charles River Laboratories, SA, Barcelona, Spain). All mice were maintained on a 12:12 h dark–light cycle at 22 ± 2 °C and were provided with tap water and a standard chow diet ad libitum. The Oviedo University Animal Care and Use Committee and the Regional Clinical Research Ethics Committee of the Principality of Asturias approved the experimental protocol. All in vivo studies were carried out according to the Spanish Government Guide and the European Community Guide for Animal Care (Council Directive 86/609/EEC).

### 2.2. Treatment

All experimental animals were subjected to a two-week acclimatization period under standard laboratory conditions before treatment. Wild-type and ob/ob mice were randomized into four experimental groups of eight animals each: non-treated control groups and melatonin-treated groups. Two hours after lights off (ZT14), melatonin (Sigma-Aldrich, St Louis, MO, USA) diluted in a minimum volume of ethanol (0.5%) was intraperitoneally injected daily under dim red light at a dose of 500 μg/kg body weight for four weeks. Non-treated animals received a comparable dose of the vehicle.

The animals were sacrificed via decapitation, and the skeletal muscle from the hind limb of each mouse was removed. Muscle samples were washed with a saline solution, immediately frozen in liquid nitrogen, and stored at 80 °C until further use. If not indicated otherwise, 0.4 g of muscle from each mouse was homogenized using an Ultra-Turrax homogenizer (Ultra-Turrax T25 digital; IKA, Staufen, Germany) in 2 mL of lysis buffer (50 mM phosphate buffer, pH 7.5, 1 mM NaF, 1 mM Na_3_VO_4_, 1 mM PMSF, and 0.1% Triton-X 100) and centrifuged for 6 min at 1500× *g* and 4 °C. Supernatants were collected, and the Bradford method [32] was used to measure protein content.

### 2.3. Macroscopic Parameters

All animals were weighed at baseline and at the end of the experiment. Upon sacrifice, the length of the lower limb perimeter and the height of each mouse were recorded. Moreover, using surgical tools, the muscle and fat mass of the lower limb were carefully removed and subsequently weighed. The body mass index (BMI), skeletal muscle index (SMI), fat mass index (FMI), and limb appendicular skeletal muscle mass index (L-ASMI) were calculated in the four experimental groups to characterize the sarcopenic muscle. Food intake was measured per cage twice a week.

### 2.4. Histological Analysis

Pieces of skeletal muscle from the hind limb were fixed via immersion in 4% formaldehyde (20,910.294, MERCK, Darmstadt, Germany), embedded in paraffin using standard methods, and cut into 5–7 μm thick sections. Sections were stained using periodic acid–Schiff (PAS) staining to evaluate muscle fiber-type patterns. In each skeletal muscle piece, four different levels separated by 300 μm were collected, and in each level, two random high-power fields (HPFs) were analyzed using a NIKON Eclipse E200 microscope (Nikon Corp., Tokyo, Japan); the size of the HPF for this microscope is 0.196 mm^2^. The proportion of type II fibers that were stained darker than type I fibers was quantified. Quantification was carried out by observers who were blinded to the genotype of the animals and treatments.

### 2.5. Mitochondrial Isolation

Mitochondrial isolation from skeletal muscle was performed following the protocol established by García-Cazarin and colleagues [33]. The tissue was washed with PBS and immediately cut slowly into small pieces for incubation in the PBS solution with 10 mM EDTA and 0.01% trypsin on ice. After 30 min of incubation, muscle pieces were transferred to mitochondrial isolation buffer 1 (10 mM EDTA, 215 mM D-mannitol, 77 mM sucrose, 20 mM HEPES, and 0.1% BSA (fatty acid-free) with pH 7.4), homogenized using a Potter–Elvehjem homogenizer and centrifuged for 10 min at 700× *g* and 4 °C. The supernatant containing cytosolic and mitochondrial fractions was transferred to a new refrigerated centrifuge tube and centrifuged again at 10,500× *g* for 10 min at 4 °C. The resulting supernatant that contained cytosolic fraction was then collected. The pellet corresponding to the mitochondrial fraction was gently resuspended in mitochondrial isolation buffer 2 (6 mM EGTA, 215 mM D-mannitol, 77 mM sucrose, 20 mM HEPES, and 0.1% BSA (fatty acid-free) with pH 7.4) and centrifuged at 10,500× *g* for 10 min at 4 °C. Finally, the supernatant was discarded, and the mitochondrial isolated pellet was resuspended in mitochondrial isolation buffer 2. The protein content in cytosolic and mitochondrial extracts was quantified using the Bradford method [32]. Tom20 was analyzed via Western blotting to ensure the purity of cytosolic and mitochondrial fractions.

### 2.6. Oxidative Stress Status

Lipid peroxidation (LPO) was studied by measuring reactive aldehyde malondialdehyde (MDA) and 4-hydroxy-2-(E)-nonenal (4-HNE) end products. The content of MDA and 4-HNE were determined in skeletal muscle homogenates using an LPO assay kit from Calbiochem (No. 437634, San Diego, CA, USA) based on the condensation of the chromogene 1-methyl-2-phenylindole with either MDA or 4-HNE [34]. Cytosolic superoxide dismutase (SOD) and mitochondrial SOD activities (EC 1.15.1.1), which catalyze the dismutation of the superoxide anion (O_2_^−^) to hydrogen peroxide (H_2_O_2_), were measured based on the inhibition of hematoxylin autoxidation to the colored compound hematein, according to the method developed by Martin and colleagues [35]. Catalase (CAT) activity was assayed in cytosolic and mitochondrial fractions using the method reported by Lubinsky and Bewley [36], which is based on the breakdown of H_2_O_2_ into O_2_ and H_2_O. Glutathione peroxidase (GSH-Px) was used to catalyze the oxidation of reduced glutathione (GSH), resulting in oxidized glutathione (GSSG) via the reduction of H_2_O_2_ to H_2_O. The GSSG produced was immediately reduced to GSH by nicotinamide adenine dinucleotide phosphate (NADPH) and glutathione reductase (GR) action. GSH-Px and GR were measured in cytosolic and mitochondrial samples, and the rate of NADPH consumption was monitored to determine both activities according to Kum-Tatt and colleagues [37].

### 2.7. Oxygen Consumption

Mitochondrial oxygen consumption was determined using a Clark-type oxygen electrode (Oxygraph, Hansatech Instruments Ltd., Pentney, UK) following the protocol developed by Silva and Oliveira [38]. Briefly, 500 μg of mitochondrial suspension was introduced into a chamber with 1 mL of mitochondrial respiration buffer (5 mM MgCl_2_, 215 mM of D-mannitol, 6.5 μM KH_2_PO_4_, 20 μM EGTA, 15 mM sucrose, 4 mM HEPES, and 0.1% BSA (fatty acid-free) with pH 7.4). Complex I and complex II respirations were measured via the addition of proper substrates and inhibitors (complex I: 10 mM glutamate + 5 mM malate, 175 nmol ADP, 1 μg oligomycin, and 1 μM carbonyl cyanide-4-(trifluoromethoxy) phenylhydrazone (FCCP); complex II: 5 mM succinate + 1 μM rotenone, 125 nmol ADP, 1 μg oligomycin, and 1 μM FCCP). The respiratory control ratio (RCR) was calculated as the ratio between maximal O_2_ consumption stimulated by ADP (State 3) and respiration in the absence of ADP or without ATP synthesis (State 4), which determines the coupling between substrate oxidation and phosphorylation [38]. ADP/O was also obtained as the amount of ADP added per amount of O_2_ consumed during State 3 [38].

### 2.8. ATP Measurement

The Adenosine 5′-triphosphate Bioluminescent Assay Kit (FLAA, Sigma-Aldrich) was used to determine ATP content in cytosolic and mitochondrial fractions. This assay measures the light emission with a luminometer based on the ATP consumption when firefly luciferase catalyzes D-luciferin oxidation.

### 2.9. Enzyme-Linked Immunosorbent Assay (ELISA)

Tumor necrosis factor alpha (TNF-α) (KMC3011, Life Technologies, Waltham, MA, USA) and interleukin 6 (IL-6) (KMC0061, Life Technologies, Waltham, MA, USA) ELISA kits were used to assess the inflammatory response in skeletal muscle tissue. All assays were performed following manufacturers’ protocols.

### 2.10. Peptide Mass Fingerprinting

Aliquots of muscle homogenate (25 μg of protein per sample) were solubilized in Laemmli sample buffer (BioRad Laboratories, Inc., Hercules, CA, USA) and boiled at 100 °C for 5 min to load onto SDS-PAGE gels. Molecular weight range markers composed of a mixture of blue-stained recombinant proteins (Precision Plus Protein All Blue Standards, BioRad) were also loaded onto the gels to identify proteins’ molecular weights. Coomassie Brilliant Blue R-250 dye (BioRad) was used to stain one-dimensional gels for protein detection. Gel images were semiquantitatively analyzed using Image Studio Lite 3.1.4 software for Macintosh (LI-COR Biosciences, Lincoln, NE, USA).

Protein bands of interest were manually excised from Coomassie-stained gels and submitted for peptide mass fingerprint identification at the Inbiotec S.L. (León, Spain) proteomics laboratory. The samples were processed and analyzed with a 4800 Proteomics Analyzer matrix-assisted laser desorption ionization time-of-flight (MALDI-TOF/TOF) mass spectrometer (ABSciex, Framingham, MA, USA) according to the previously described methods of Oliván et al. [39]. A database search on Mascot Generic Files combining MS and MS/MS spectra was performed using Mascot v 2.2 from Matrix Science through the Global Protein Server v 3.6 (ABSciex). When the Mascot score was greater than 85 points, the identified protein was considered a valid candidate.

### 2.11. Western Blotting

Muscle tissue homogenates (50–100 μg of protein per sample) were denaturalized at 100 °C for 5 min in a Laemmli buffer (BioRad) and then separated via electrophoresis in SDS-PAGE gel at 200 V and transferred to a polyvinylidene fluoride membranes (Immobilon TM-P; Millipore Corp., Burlington, MA, USA) at 350 mA. After blocking membranes for 1 h at room temperature with 10% (*w*/*v*) nonfat dry milk in TBS (50 mM Tris-HCl, (pH 7.5) and 150 mM NaCl), the membranes were incubated overnight at 4 °C with the respective primary antibodies: AIF (sc-13116; Santa Cruz Biotechnology, Dallas, TX, USA); ATF6α (sc-22799, Santa Cruz Biotechnology); BAX (2772, Cell Signaling, Danvers, MA, USA); Beclin-1 (4445, Cell Signaling); CaMKII (3362, Cell Signaling); CHOP (L63F7) (2895, Cell Signaling); CI-20 (NDUFB8) (ab110242, Abcam, Cambridge, UK); CII-30 (SDHB) (ab14714, Abcam); CIII-Core II (UQCRC2) (ab14745, Abcam); CIV-I (MTCO1) (ab14705, Abcam); CV-a (ATP5A) (ab14748, Abcam); cyclophilin D (ab110324 (MSA04), Abcam); cytochrome C (ab110252, Abcam); DRP1 (D6C7) (8570S, Cell Signaling); eIF2α (5324, Cell Signaling); hexokinase-II (2867; Cell Signaling); IRE1α (3294, Cell Signaling); LC3 (PD014, Medical and Biological Laboratories Co., LDT, Tokyo, Japan); MFN1 (sc-50330; Santa Cruz Biotechnology); MFN2 (D2D10) (9482S, Cell Signaling); MURF1 (ab77577; Abcam); p62 (H00008878-M01, Abnova, Taipe, Taiwan); p70S6K (9202; Cell Signaling); phospho-AKT (9271, Cell Signaling); phospho-CaMKII (3361, Cell Signaling); phospho-eIF2α (3398, Cell Signaling); phospho-mTOR (5536; Cell Signaling); phospho-p70S6K (9206; Cell Signaling); phospho-RYR1 (ab59225, Abcam); PI3K (4255; Cell Signaling); porin (MSA03) (ab14734, Abcam); XBP1 (sc-8015; Santa Cruz Biotechnology). Each antibody was previously diluted 1:1000 in TBS containing 2% (*w*/*v*) nonfat dry milk. After three 10 min washes in TBS-T (TBS containing 0.1% Tween-20), the membranes were incubated with the corresponding horseradish peroxidase-conjugated secondary antibody (Sigma-Aldrich, St Louis, MO, USA) diluted 1:10,000 in TBS containing 1% (*w*/*v*) nonfat dry milk for 1 h at room temperature. After three 10 min washes in TBS-T, the membranes were developed using a chemiluminescent horseradish peroxidase substrate (WBKLS0500, Millipore Corp., Darmstadt, Germany) according to the manufacturer’s protocol. Densities of protein bands were analyzed quantitatively with Image Studio Lite 3.1.4 software (LI-COR Biosciences, Lincoln, NE, USA). Variations in the levels of the typical housekeeping proteins (GAPDH, β-actin, and α-tubulin) were observed, so Ponceau S staining was used to ensure equal protein loading in tissue homogenates [40]. As OXPHOS levels were studied in muscle homogenates, we normalized these data with Ponceau S staining and with porin to evaluate respiratory chain complexes per mitochondria. Levels of hexokinase-II, BAX, AIF, and cytochrome C were normalized with porin in the mitochondrial fraction and with Ponceau S staining in the tissue homogenate.

### 2.12. RNA Extraction and RT-qPCR Analysis

RNA was isolated from mouse skeletal muscle tissue using TRI reagent (T9424, Sigma-Aldrich). Total RNA extraction levels were quantified using NANO DROP 2000 (Thermo Fisher Scientific, Waltham, MA, USA). A high-capacity cDNA reverse transcription kit (4,368,814, Applied Biosystems, Foster City, CA, USA) was used to synthesize complementary DNA (cDNA) from total RNA extracts following the manufacturer’s instructions. Expression levels of genes were determined in the StepOne Real-Time PCR System (Applied Biosystems) with quantitative real-time PCR (RT-qPCR) reactions using Power SYBR Green PCR Master Mix (4,367,659; Applied Biosystems) and the following specific pairs of primers: *Acaca*, forward: 5′-AATGGCATTGCAGCAGTGAA-3′, reverse: 5′-CACATAGTGATCTGCCATCTTAATGTATT-3′; *Adipor1*, forward: 5′-CCCACCATGCCATGGAGA-3′, reverse: 5′-GCCATGTAGCAGGTAGTCGTTGT-3′; *Adipor2*, forward: 5′-CAGGAAGATGAGGGCTTTATGG-3′, reverse: 5′-GAGGAAGTCATTATCCTTGAGCCA-3′; *Atp2a1*, forward: 5′-CTGACCGCAAGTCAGTGCAA-3′, reverse: 5′-GGATGGACTGGTCAACCCG-3′; *Calstabin1*, forward: 5′-GGGGATGCTTGAAGATGGAA-3′, reverse: 5′-TTGGCTCTCTGACCCACACTC-3′; *Gapdh*, forward: 5′-CAATGACCCCTTCATTGACC-3′, reverse: 5′-TGGAAGATGGTGATGGGATT-3′; *Insr*, forward: 5′-TGAACGCCAAGAAGTTTGTG-3′, reverse: 5′-CAGCCAGGCTAGTGATTTCC-3′; *Jnk*, forward: 5′-GATTGGAGATTCTACATTCACAG-3′, reverse: 5′-CTTGGCATGAGTCTGATTCTGAA-3′; *Lamp2a*, forward: 5′-GAAGTTCTTATATGTGCAACAAAGAGCAG-3′, reverse: 5′-CTAAAATTGCTCATATCCAGCATGATG-3′; *Ppargc1a*, forward: 5′-GACTTGGATACAGACAGCTTTCTGG-3′, reverse: 5′-GCTAGCAAGTTTGCCTCATTCTCT-3′; *Ppara*, forward: 5′-TGAAGAACTTCAACATGAACAAG-3′, reverse: 5′-TTGGCCACCAGCGTCTTC-3′; *Pparg*, forward: 5′-ACTATGGAGTTCATGCTTGTGAAGGA-3′, reverse: 5′-TTCAGCTGGTCGATATCACTGGAG-3′; *Ryr1*, forward: 5′-AAGGCGAAGACGAGGTCCA-3′, reverse: 5′-TTCTGCGCGTTGCTGTGG-3′; *Tfam*, forward: 5′-ACCTCGTTCAGCATATAACGTTTATGTA-3′, reverse: 5′-GCTCTTCCCAAGACTTCATTTCAT-3′; *Xbp1*, forward: 5′-GAGGAGAAGGCGCTGAGGA-3′, reverse: 5′-CCTCTTCAGCAACCAGGGC-3′. Thermocycling conditions were as follows: a holding stage for 10 min at 95 °C; then a cycling stage of 40 15 s cycles at 95 °C and 1 min at 60 °C; and finally a 15 s melt curve stage at 95 °C, 1 min at 60 °C and 15 min at 95 °C. The average cycle threshold (Ct) value at which each gene was detectable was calculated. The Ct of the *Gapdh* was used for normalization. Relative changes in gene expression levels were determined using the 2^−ΔΔCt^ method [41]. All primers were intron-exon spanning according to the data obtained from the Ensembl genome database project: www.ensembl.org (accessed on 2 November 2023). The specificity of PCR primers was assessed with previous analysis using the program blast: www.blast.ncbi.nlm.nih.gov (accessed on 2 November 2023) to discard the annealing of the primers to other genes from Mus musculus different from the target one and through the melting curve analysis of PCR reaction products. The gene accession number is available in Table 1.

### 2.13. Statistical Analysis

The SPSS statistical software package 20.0.0 (SPSS Inc., Chicago, IL, USA) and GraphPad Prism 6.0 (GraphPad Software, Inc., La Jolla, CA, USA) for Macintosh were used for all statistical analyses and graph design. Data are mean values ± standard deviation of the mean (SD). All the experiments were performed with 8 mice (*n* = 8) per experimental group. The normality of the data was analyzed using the Kolmogorov–Smirnov test. Since all data had normal distribution, they were analyzed with a parametric two-way ANOVA followed by Bonferroni post hoc test. A *p* < 0.05 was considered statistically significant.

## 3. Results

### 3.1. Leptin Deficiency Reduces Skeletal Muscle Mass

A two-fold increase in weight was observed in leptin-deficient mice, compared with wild-type mice; however, their muscle weight was reduced by 27%. Ob/ob mice also showed higher FMI and BMI. Interestingly, ob/ob mice exhibited increased FMI and reduced SMI and L-ASMI compared with wild-type mice, indicating the coexistence of higher fat mass, particularly intramuscular and peripheral fat, with decreased musculoskeletal mass (Table S1). Furthermore, the color of skeletal muscle shifted from dark red in wild-type mice to pale red in ob/ob mice, suggesting increased fat infiltration (Figure S1). Melatonin treatment did not affect these parameters.

### 3.2. Melatonin Remodels the Mitochondrial Respiration Affected by Leptin Deficiency

**Figure 1:**
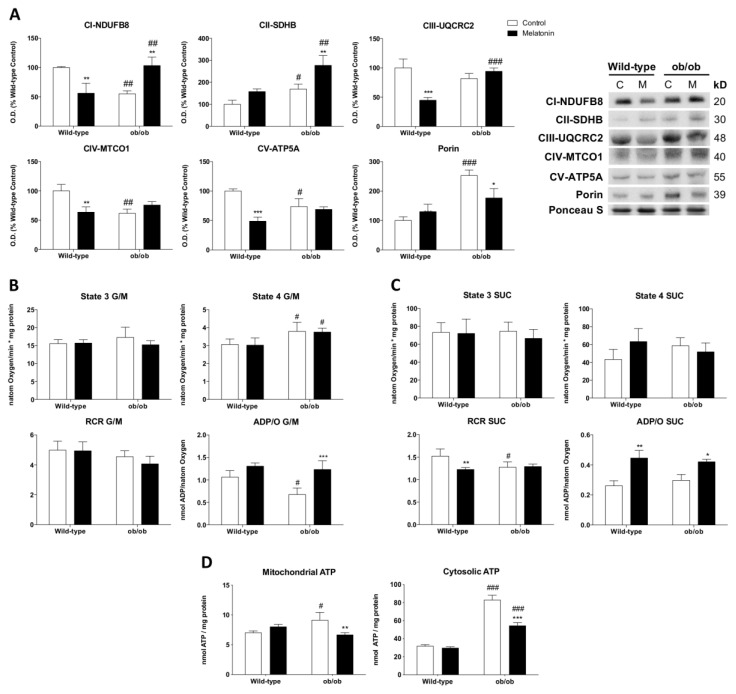
Melatonin remodels OXPHOS complexes and oxidative phosphorylation in skeletal muscle from ob/ob mice: (**A**) Protein expression analysis of OXPHOS subunits from each complex (NDUFB8, SDHB, UQCRC2, MTCO1, and ATP5A) and porin. Data are mean of optical density (O.D.) expressed as a percentage of wild-type control mice. Porin and ponceau staining were used as loading control. (**B**,**C**) ADP-stimulated O_2_ consumption (State 3), respiration in the absence of ADP-stimulation (State 4), respiratory control ratio (RCR), and ADP/O were analyzed using glutamate/malate (G/M) and succinate (SUC) as respiration substrates of complexes 1 and 2, respectively. Data are mean ± SD. (**D**) Basal mitochondrial and cytosolic ATP content. Data are mean ± SD. Histograms show Wild-type and ob/ob mice in white and melatonin-treated mice in black. Statistical comparisons: # wild-type vs. ob/ob; * control vs. melatonin. The main effects of leptin deficiency and melatonin treatment were detected via a two-way ANOVA (*n* = 6). The number of symbols marks the level of statistical significance: one for *p* < 0.05, two for *p* < 0.01, and three for *p* < 0.001.

To investigate how the absence of leptin impacts mitochondria, we first studied the levels of porin-normalized OXPHOS subunits in muscle homogenates to evaluate the respiratory chain content per mitochondria. Leptin-deficient mice presented alterations in mitochondrial electron transport chain (ETC) machinery, characterized by a reduced protein expression of subunits from complexes I (NDUFB8), IV (MTCO1), and V (ATP5A) and increased levels of subunits from complex II (SDHB). The increased expression of the mitochondrial mass marker porin in ob/ob mice suggests higher amounts of mitochondria than in wild-type mice. Melatonin administration in ob/ob mice significantly increased NDUFB8 and SDHB subunits and reduced porin expression (Figure 1A). Then, we studied mitochondrial respiration rates in isolated mitochondria from skeletal muscles. According to data obtained from State 3 using glutamate/malate as respiration substrates, there were no differences in the ADP-stimulated respiration among all the groups. However, ob/ob mice showed higher proton leak-dependent respiration (State 4). The RCR, which indicates the mitochondrial coupling state, decreased slightly. Melatonin injection did not induce any change in State 4 respiration and RCR in both genotypes. Nevertheless, the effectiveness of oxidative phosphorylation (ADP/O) in the presence of glutamate/malate was lower in ob/ob animals, an effect reverted by melatonin (Figure 1B). Conversely, the mitochondria of succinate-energized ob/ob mice in the presence of rotenone (commonly used to inhibit complex I) showed preserved State 4 and ADP/O. Hence, respiration from complex II in obese animals maintained the effectiveness of oxidative phosphorylation in spite of a slight decrease in RCR. In this case, melatonin was also able to increase ADP/O in ob/ob animals (Figure 1C). Additionally, obese mice showed significantly higher mitochondrial and cytosolic ATP levels than those from wild-type mice. Melatonin administration restored mitochondrial ATP and reduced cytosolic ATP (Figure 1D).

### 3.3. Melatonin Reduces Leptin-Deficiency-Induced Oxidative Damage with a Non-Mitochondrial Origin through the Regulation of Cytosolic Antioxidant Enzymes

**Figure 2:**
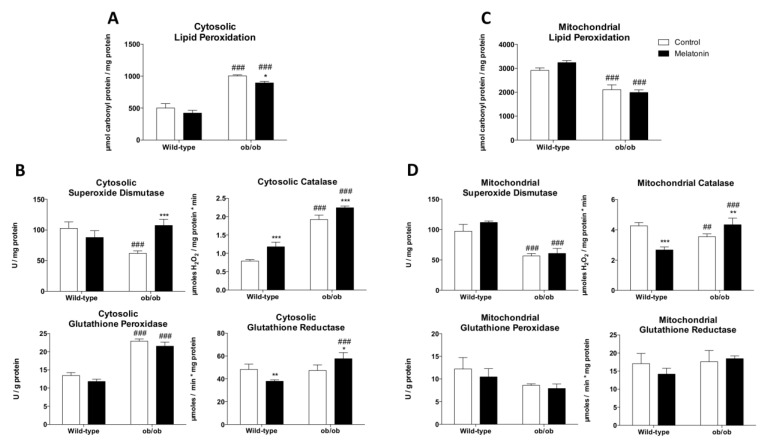
Melatonin reduces oxidative stress-induced cytosolic damage and enhances antioxidant defense in skeletal muscle from ob/ob mice: (**A**) Determination of cytosolic lipid oxidative damage (lipid peroxidation). Data are mean ± SD. (**B**) Antioxidant system evaluation by determining the activity of antioxidant enzymes (superoxide dismutase, catalase, glutathione peroxidase, and glutathione reductase) in cytosolic fraction. Data are mean ± SD. (**C**) Determination of mitochondrial lipid oxidative damage (lipid peroxidation). Data are mean ± SD. (**D**) Antioxidant system evaluation by determining the activity of antioxidant enzymes (superoxide dismutase, catalase, glutathione peroxidase, and glutathione reductase) in the mitochondrial fraction. Data are mean ± SD. Histograms show Wild-type and ob/ob mice in white and melatonin-treated mice in black. Statistical comparisons: # wild-type vs. ob/ob; * control vs. melatonin. The main effects of leptin deficiency and melatonin treatment were detected using a two-way ANOVA (*n* = 6). The number of symbols marks the level of statistical significance: one for *p* < 0.05, two for *p* < 0.01, and three for *p* < 0.001.

The cytosolic fraction of skeletal muscle from ob/ob mice manifested higher lipid peroxidation levels and imbalanced antioxidant defense as evidenced by low SOD and high CAT and GSH-Px activities (Figure 2A,B). Moreover, leptin deficiency triggered an inflammatory response characterized by increased IL-6 and TNF-α levels (Figure S2). As enhanced mitochondrial proton leak is considered an adaptive metabolic response to reduce mitochondrial reactive oxygen species (ROS) production and limit oxidative damage [42], we also evaluated oxidative stress status in isolated mitochondria. Concordantly, we found that leptin deficiency lowered mitochondrial lipid peroxidation levels and SOD and CAT activities (Figure 2C,D). Melatonin administration reduced cytosolic lipid peroxidation levels by increasing cytosolic SOD, CAT, and GR activities, while mitochondrial oxidative damage remained unaltered.

### 3.4. Melatonin Relieves the Altered Coupling between Glycolysis and Mitochondrial Respiration Caused by Leptin Deficiency

**Figure 3:**
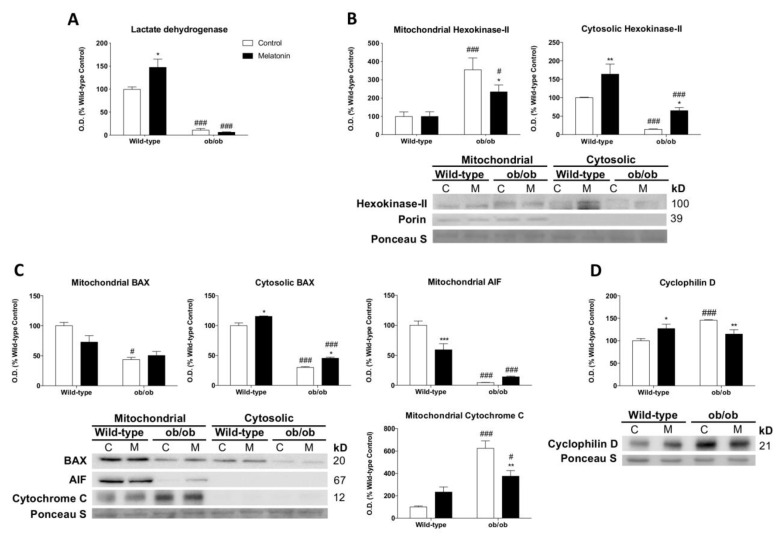
Melatonin regulates mitochondria-bound hexokinase-II and apoptosis response in skeletal muscle from ob/ob mice: (**A**) Lactate dehydrogenase identification via laser desorption/ionization-time of flight (MALDI-TOF/TOF) mass spectrometry and protein level analysis. Data are mean of optical density (O.D.) ± SD expressed as a percentage of wild-type control mice. (**B**) Expression of hexokinase-II in mitochondrial and cytosolic fractions. Data are mean of optical density (O.D.) ± SD expressed as a percentage of wild-type control mice. Ponceau staining was used as the loading control. (**C**) Protein expression analysis of BAX, AIF, and cytochrome c to evaluate apoptosis showed significant changes in cytosolic and isolated mitochondria extracts. Ponceau staining was used as the loading control. (**D**) Cyclophilin D protein content showed significant changes in mPTP stabilization. Data are expressed in optical density (O.D.) as a percentage of wild-type control mice. Ponceau staining was used as the loading control. Histograms show Wild-type and ob/ob mice in white and melatonin-treated mice in black. Statistical comparisons: # wild-type vs. ob/ob; * control vs. melatonin. The main effects of leptin deficiency and melatonin treatment were detected using a two-way ANOVA (*n* = 6). The number of symbols marks the level of statistical significance: one for *p* < 0.05, two for *p* < 0.01, and three for *p* < 0.001.

Next, we evaluated the influence of melatonin on hexokinase-II (HK-II), since this enzyme binds to mitochondria to diminish ROS production and maintain the aerobic metabolism of glucose [43]. Our results showed downregulated lactate dehydrogenase protein expression and higher levels of mitochondria-bound HK-II (mtHK-II) in leptin-deficient mice, indicating a tight coupling of glycolysis with mitochondrial respiration (Figure 3A,B). HK-II is also considered a control point of apoptosis [44]. Mitochondria from ob/ob mice showed lower levels of Bcl-2-associated X protein (BAX) and apoptosis-inducing factor (AIF) accompanied by increased mitochondrial cytochrome c expression, suggesting a lower susceptibility to events triggering mitochondrial outer membrane permeabilization (Figure 3C). Likewise, obese mice exhibited overexpressed cyclophilin D (Figure 3D), a regulatory component of the mPTP that exerts apoptosis-suppressing effects mediated by mtHK-II. Interestingly, melatonin increased cytosolic HK-II and reduced mtHK-II, cyclophilin D, and mitochondrial cytochrome c expression in ob/ob mice. BAX and AIF levels remained unaltered.

### 3.5. Melatonin Modulates Lipid and Glucose Metabolism in ob/ob Mice

As the transcriptional activation of peroxisome proliferator-activated receptor-alpha (*Ppara*) is fundamentally important for energy and lipid metabolism, we explored whether leptin deficiency affects fatty acid oxidation and synthesis. Obese mice showed no major changes in mRNA levels of adiponectin receptors 1 (*Adipor1*) and 2 (*Adipor2*). Interestingly, we found that *Ppara* mRNA expression was remarkably increased, suggesting that β-oxidation is activated upon leptin deficiency. The mRNA levels of peroxisome proliferator-activated receptor-gamma (*Pparg*) remained unaltered. Obese mice also showed increased mRNA expression of acetyl-CoA carboxylase (*Acaca*) (Figure S3A), which is a key regulatory step of lipogenesis. To further investigate if *Ppara*-altered-response could drive the development of insulin resistance and glucose intolerance, the c-Jun N-terminal kinase (*Jnk*) cascade was analyzed. Obese mice exhibited increased mRNA levels of insulin receptor (*Insr*). However, the leptin deficiency triggered *Jnk* expression in muscle fibers (Figure S3B). Although melatonin treatment led to higher *Acaca* expression in ob/ob mice, its administration increased *Pparg* levels, which have been described to be closely involved in the stimulation of glucose uptake [45].

### 3.6. Melatonin Remodels Mitochondrial Biogenesis and Enhances mnf2-Dependent Mitochondrial Fusion upon Leptin Deficiency

**Figure 4:**
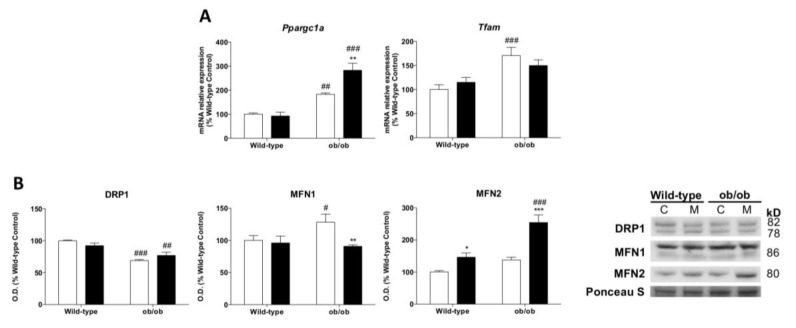
Melatonin modulates mitochondrial biogenesis and mitochondrial dynamics in skeletal muscle from ob/ob mice: (**A**) Relative mRNA expression of mitochondrial biogenesis genes (*Tfam* and *Ppargc1a*). Data are mean of mRNA relative expression ± SD expressed as a percentage of wild-type control mice. (**B**) Protein expression analysis of mitochondrial remodeling markers (DRP1, MFN1, and MFN2). Data are mean of optical density (O.D.) ± SD expressed as a percentage of wild-type control mice. Ponceau staining was used as the loading control. Histograms show Wild-type and ob/ob mice in white and melatonin-treated mice in black. Statistical comparisons: # wild-type vs. ob/ob; * control vs. melatonin. The main effects of leptin deficiency and melatonin treatment were detected using a two-way ANOVA (*n* = 8). The number of symbols marks the level of statistical significance: one for *p* < 0.05, two for *p* < 0.01, and three for *p* < 0.001.

To further investigate whether leptin-deficiency-induced obesity may underlie other mitochondrial adaptations, we studied mitochondrial biogenesis and dynamics. Leptin-deficient mice showed higher mRNA levels of peroxisomal proliferator-activated receptor coactivator 1 alpha (*Ppargc1a*) and mitochondrial transcription factor A (*Tfam*), suggesting the induction of mitochondrial biogenesis (Figure 4A). Our results also revealed important changes in the molecular machinery of mitochondrial dynamics, showing the depletion of fission protein dynamin-related protein 1 (DRP1) and the upregulation of the fusion protein mitofusin 1 (MFN1) in ob/ob animals. However, mitofusin 2 (MFN2) remained unaltered (Figure 4B). Melatonin treatment restored *Tfam* levels in leptin-deficient mice but increased the expression of *Ppargc1a*. Melatonin showed no effects on DRP1 content in ob/ob animals. Intriguingly, melatonin downregulated MFN1 but upregulated MFN2 protein.

### 3.7. Melatonin Modulates the Leptin-Deficiency-Induced Deregulation of UPR

**Figure 5:**
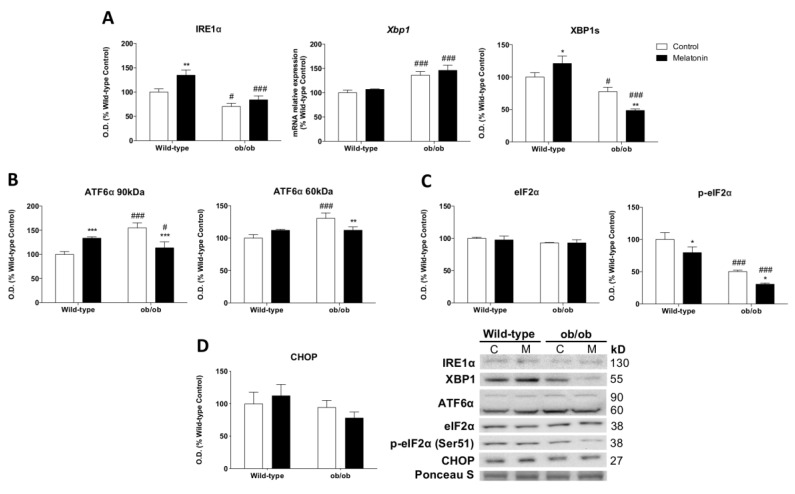
Melatonin regulates unfolded protein response (UPR) in skeletal muscle from ob/ob mice: (**A**) Protein and relative mRNA expression of markers involved in the UPR branch activated by IRE1α and the specific splicing of *Xbp1* (IRE1α, *Xbp1*, and XBP1s). Data are mean of optical density (O.D.) or mRNA relative expression ± SD expressed as a percentage of wild-type control mice. Ponceau staining was used as the loading control. (**B**) The UPR signaling branch activated via a specific proteolytic cleavage of ATF6α was analyzed. Data are mean of optical density (O.D.) ± SD expressed as a percentage of wild-type control mice. Ponceau staining was used as the loading control. (**C**,**D**) UPR signaling markers from the branch activated by eIF2α phosphorylation (eIF2α, p-eIF2α, and CHOP) were analyzed. Data are mean of optical density (O.D.) ± SD expressed as a percentage of wild-type control mice. Ponceau staining was used as the loading control. Histograms show Wild-type and ob/ob mice in white and melatonin-treated mice in black. Statistical comparisons: # wild-type vs. ob/ob; * control vs. melatonin. The main effects of leptin deficiency and melatonin treatment were detected using a two-way ANOVA (*n* = 8). The number of symbols marks the level of statistical significance: one for *p* < 0.05, two for *p* < 0.01, and three for *p* < 0.001.

Since the unfolded protein response (UPR) regulates pro-survival and pro-death mechanisms in an attempt to restore metabolic homeostasis [46], additional analyses were conducted to study the three UPR arms. Obese mice exhibited reduced inositol-requiring enzyme 1 alpha (IRE1α) protein levels and increased X-box binding protein 1 (*Xbp1*) mRNA expression. The functionally active transcription factor XBP1s resulting from the spliced *Xbp1* mRNA showed a decline (Figure 5A). These results indicate that the IRE1α/XBP1 pathway, which is the point of confluence of endoplasmic reticulum (ER) stress, insulin signaling, and inflammation [47], was deactivated in leptin-deficient mice. The activation of the transcription factor 6 alpha (ATF6α) pathway was increased in ob/ob animals (Figure 5B), indicating the activation of adaptive responses against the stress induced by the accumulation of unfolded/misfolded proteins in the ER. However, the phosphorylation at Ser51 of the translation initiation factor alpha (eIF2α), which is involved in the reduction in global protein synthesis [48], was deactivated in ob/ob mice (Figure 5C). Nevertheless, the C/EBP homologous protein (CHOP), a key mediator of the ER stress-mediated apoptosis pathway, showed no differences between the groups (Figure 5D). Melatonin treatment restored the ATF6α pathway and induced an even greater deactivation of IRE1α/XBP1 and eIF2α pathways. Therefore, melatonin in skeletal muscle from ob/ob mice seems to regulate protein homeostasis and/or UPR adaptative responses.

### 3.8. Melatonin Attenuates the Leptin-Deficiency-Induced Activation of Autophagy

**Figure 6:**
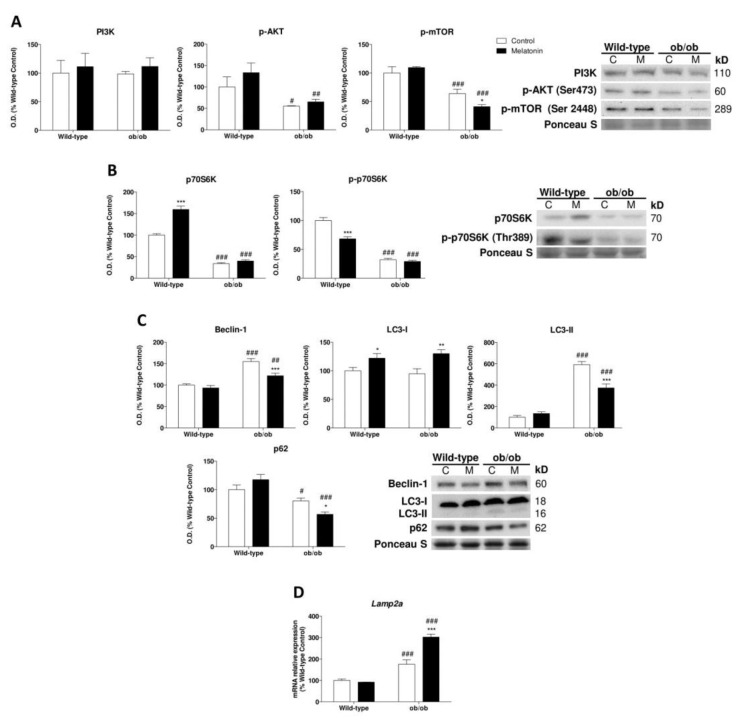
Melatonin partially restores proteostasis through autophagy regulation in skeletal muscle from ob/ob mice: (**A**) PI3K, p-AKT, and p-mTOR protein content show significant changes between genotype remodeling protein synthesis and autophagy responses. Data are mean of optical density (O.D.) ± SD expressed as a percentage of wild-type control mice. Ponceau staining was used as the loading control. (**B**) Protein expression analysis of markers downstream of protein biosynthesis pathway (p70S6K and p-p70S6K). Data are mean of optical density (O.D.) ± SD expressed as a percentage of wild-type control mice. Ponceau staining was used as the loading control. (**C**) Protein levels of autophagy mechanism (Beclin-1, LC3-I, LC3-II, and p62). Data are mean of optical density (O.D.) ± SD expressed as a percentage of wild-type control mice. Ponceau staining was used as the loading control. (**D**) Relative mRNA expression of *Lamp2a* implicated in chaperone-mediated autophagy analysis to evaluate protein removal upon mild oxidative stress. Data are mean ± SD expressed as a percentage of wild-type control mice. Histograms show Wild-type and ob/ob mice in white and melatonin-treated mice in black. Statistical comparisons: # wild-type vs. ob/ob; * control vs. melatonin. The main effects of leptin deficiency and melatonin treatment were detected via a two-way ANOVA (*n* = 8). The number of symbols marks the level of statistical significance: one for *p* < 0.05, two for *p* < 0.01, and three for *p* < 0.001.

We then further explored how melatonin regulates proteostasis upon leptin deficiency. Ob/ob mice showed no major changes in phosphoinositide-3-kinase (PI3K) protein expression and a lower phosphorylated protein expression of protein kinase B (AKT) and mammalian target of rapamycin (mTOR) at Ser473 and Ser2448 (Figure 6A). The ribosomal protein S6 kinase (p70S6K) phosphorylated at Thr389 was also reduced (Figure 6B), indicating protein synthesis suppression. Then, we evaluated the activation of the lysosomal-autophagy protein degradation system. We found increased Beclin-1 and microtubule-associated protein 1 light chain 3 (LC3) converted to autophagosome-associating protein (LC3-II) and decreased content of the ubiquitin-binding protein p62 (p62) in ob/ob animals, indicating a higher autophagic response (Figure 6C). Moreover, chaperone-mediated autophagy (CMA), determined by lysosome-associated membrane protein type 2a (*Lamp2a*) mRNA levels, was increased in ob/ob mice (Figure 6D). Thus, leptin deficiency breaks protein turnover and increases proteolysis. Interestingly, melatonin treatment regulated autophagic response in ob/ob mice through a reduction in Beclin-1 and LC3-II expression while maintaining the low levels of p62. Furthermore, CMA response was triggered by melatonin in comparison to untreated obese animals. These data indicate that melatonin partly restores proteostasis.

### 3.9. Melatonin Inhibits the Leptin-Deficiency-Induced Glycolytic-to-Oxidative Myofiber-Type Switch and Improves Muscle Excitation–Contraction Coupling

**Figure 7:**
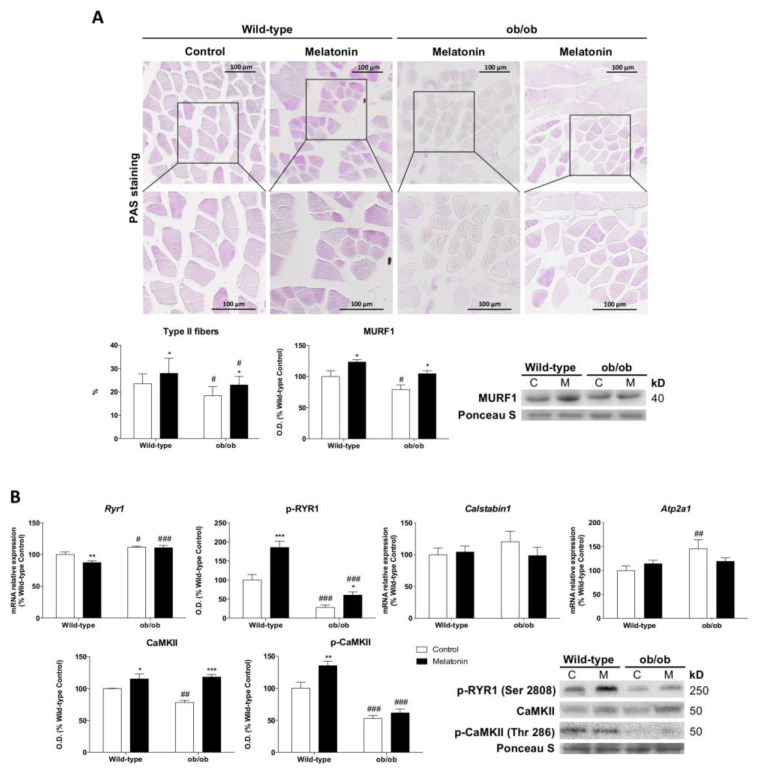
Melatonin restores fiber-type proportion and improves muscle excitation–contraction coupling in skeletal muscle from ob/ob mice: (**A**) Light microscopy images of periodic acid–Schiff (PAS)-stained sections from skeletal muscle tissue and the determination of the proportion of darker-stained type II fibers. The boxes represent the area of the images shown at higher magnification. Scale bars: 100 μm. Protein expression of MURF1 is related to an increased proportion of type II fibers. Data from protein expression analysis are mean of optical density (O.D.) ± SD expressed as a percentage of wild-type control mice. Ponceau staining was used as the loading control. (**B**) Relative mRNA and protein expression of markers involved in the skeletal muscle excitation–contraction pathway (*Ryr1*, p-RYR1, *Calstabin1*, *Atp2a1*, CaMKII, and p-CaMKII). Data are mean of mRNA relative expression or optical density (O.D.) ± SD expressed as a percentage of wild-type control mice. Ponceau staining was used as the loading control. Histograms show Wild-type and ob/ob mice in white and melatonin-treated mice in black. Statistical comparisons: # wild-type vs. ob/ob; * control vs. melatonin. The main effects of leptin deficiency and melatonin treatment were detected using a two-way ANOVA (*n* = 8). The number of symbols marks the level of statistical significance: one for *p* < 0.05, two for *p* < 0.01, and three for *p* < 0.001.

We found a functional muscle fiber-type switching toward the formation of oxidative type I fibers. Quantitative studies of PAS muscle-stained sections showed a smaller percentage of glycolytic type II fibers in ob/ob mice. Consistently, the protein expression of muscle ring-finger protein 1 (MURF1), which regulates type II fiber trophicity, was reduced in ob/ob mice, confirming that the lack of leptin favors oxidative fibers (Figure 7A). Furthermore, the skeletal muscle of leptin-deficient mice showed reduced activation of the ryanodine receptor 1 (RYR1) channel via phosphorylation. P(Ser2808)-RYR1 depletion along with decreased phosphorylated Ca^2+^/calmodulin-dependent protein kinase II (CaMKII) at Thr286 indicate the inhibition of calcium release via sarcoplasmic reticulum (Figure 7B). Melatonin treatment restored the percentage of type II fibers and increased P(Ser2808)-RYR1, CaMKII, and P(Thr286)-CaMKII levels slightly improving the excitation-contraction coupling.

## 4. Discussion

It has become increasingly clear that leptin seems to be essential for skeletal muscle growth and maintenance [49], and in fact, our results revealed severe skeletal muscle loss in leptin-deficient mice. Recent studies showed that leptin contributes to cellular growth by promoting mitochondrial metabolism [11,50]. However, the primary mechanisms underlying impaired leptin signaling-associated skeletal muscle wasting remain unknown. Our results demonstrate that leptin deficiency induces changes in mitochondrial energy metabolism probably associated with the augmented fatty acid β-oxidation. Increased proton leak respiration in isolated muscle mitochondria from ob/ob mice indicates an incomplete coupling of substrate oxygen and ATP generation. Fatty acid overload was demonstrated to activate uncoupling proteins and proton leak, as well as mitochondrial biogenesis, as a protective strategy against oxidative damage [51,52]. We found that leptin deficiency upregulated *Ppara* signaling and mitochondrial biogenesis and reduced mitochondrial oxidative damage and mitochondrial antioxidant defense, supporting increased fatty acid oxidation and mitochondrial adaptation. Interestingly, signs of increased lipid peroxidation were observed in the cytosolic fraction of skeletal muscle from ob/ob mice. Our work leads us to speculate that cytosolic oxidative damage is derived from peroxisomal fatty acid oxidation. In support of this possibility, evidence shows that chronic exposure to fatty acids leads to increased peroxisomal H_2_O_2_ generation and oxidative damage [53,54]. Despite the mitochondrial adaptation, this study reveals that leptin deficiency reflects cytosolic muscle damage, which may be caused by altered lipid metabolism. This verifies that the lack of leptin promotes the oxidation of fatty acids at the peroxisomal level, contributing to increased muscle damage, and therefore it is one of the future challenges in this area of research.

Recently, HK-II has emerged as a powerful regulator of metabolic and cell survival pathways. Notably, mtHK-II favors mitochondrial uncoupling to suppress mitochondrial ROS and maintain aerobic respiration in fed mice and high-glucose-treated cells [43]. However, there is no evidence in the literature that allows us to speculate about the role of leptin in the HK-II regulation. Here, we found for the first time that leptin deficiency not only promotes mtHK-II but also critically reduces enzymes for lactic acid fermentation, thereby coupling glycolysis to oxidative phosphorylation. In addition, mtHK-II regulates apoptosis by interfering with the ability of Bax to bind to mitochondria and therefore inhibits mitochondrial outer membrane permeabilization [44,55]. Although recent works have not studied the effect of leptin on HK-II modulation, they have shown alterations in the apoptotic response. Leptin was found to trigger apoptosis in adipocytes [56] but inhibit the stress-induced apoptosis of T lymphocytes [57]. In skeletal muscle, we found evidence indicating that leptin deficiency reduced mitochondrial Bax and increased cyclophilin D, inhibiting mitochondrial cytochrome c and AIF release. It was previously described that mitochondrial AIF deficiency and augmented cytochrome c reduce ROS generation [58,59]. Therefore, our results highlight the finding that the leptin-deficiency-related reduction in AIF and increase in cytochrome c indicate mitochondrial protection against ROS through HK-II regulation. Moreover, mitochondrial dynamics are also essential in regulating metabolism and cell fate. Mitochondrial fusion in respiratory active cells allows for the spreading of metabolites and enzymes, while mitochondrial fission contributes to apoptosis [60]. The leptin-deficiency-induced metabolic remodeling stimulates mitochondrial fusion to optimize mitochondrial function and evade cell-death-related events.

Importantly, our results also provide evidence that the leptin-deficiency-induced set of metabolic derangements compromises cellular quality control mechanisms. Previous studies have reported that the UPR is activated in chronic metabolic diseases such as obesity [61,62]. Moreover, it was also found that the genetic imposition of diminished ER capacity leads to severe leptin resistance and obesity development [63]. Indeed, another study revealed that leptin treatment is able to restore basal levels of ER stress [64]. Here, we revealed the mechanism underlying the exact regulation of leptin deficiency in the three arms of the UPR. We observed that the lack of leptin also promotes the deactivation of the UPR. In particular, leptin deficiency deactivated the IRE1α/XBP1 and eIF2α pathways, indicating reduced accumulation of misfolded proteins. ATF6α, which is implicated in lipid biosynthesis, was the only UPR arm activated. In accordance with our results indicating lipid anabolism promotion, this leptin-deficiency-related ATF6α activation could contribute to the significant abundance of cellular lipids. Therefore, leptin appears to be a key factor involved in ER stress. It was also found that autophagy negatively regulates the UPR [65]. In recent years, research has speculated about the potential influence of leptin on autophagy; however, these findings remain disputed. Leptin was found to induce autophagy in adipocytes [17], while it appeared to suppress this response in chondrocytes [18]. Our work provided evidence indicating that leptin-deficient mice stimulated autophagy and downregulated protein anabolism via the inhibition of the AKT/mTOR pathway in skeletal muscle, which was likely a consequence of the regulatory role of HK-II on the nutrient-sensing mTOR pathway. Upon glucose deprivation, HK-II inhibits mTOR, thus preserving cellular integrity [66]. Leptin deficiency induces a starvation signal and, in response to the feeling of limited energy, reserves ob/ob mice increased mtHK-II levels, supporting autophagy stimulation through mTOR inhibition and energy storage. Additionally, this response enhances protein turnover, which significantly diminishes misfolded protein accumulation and deactivates IRE1α/XBP1 and eIF2α arms of the UPR.

Lastly, we found that leptin deficiency has a major impact on muscle quality, driving the functional fiber-type switch toward the formation of oxidative type I. Since mitochondrial uncoupling triggers the formation of fiber-type I, which is related to sarcopenic muscle [67,68], leptin-deficiency-induced metabolic remodeling may cause muscle aging and contribute to the muscle atrophy observed in this study. The suppression of excitation–contraction coupling in skeletal muscles of ob/ob mice likely occurs due to defective myofiber protein assembly, combined with the fact that slow-twitch muscle fibers contract with lower force [69]. These results also indicate that leptin deficiency leads to higher cellular ATP pools due to slower consumption by the contractile apparatus.

Melatonin and leptin display daily rhythms that may contribute to fuel harvesting and energy homeostasis due to the strong interplay between circadian and metabolic systems [23,24,25]. However, the decreased amplitude of the nocturnal pineal melatonin peak has been described in obese rodents [70], and altered leptin production has also been attributed to a lack of circadian control, having an impact on appetite and energy balance [24,25]. Indeed, melatonin deficiency has been demonstrated to correlate with obesity [71,72], and the absence of melatonin leads to leptin resistance [65], making leptin treatments ineffective. Then, one can ask whether melatonin supplementation at night normalizes some of the obesity-related metabolic alterations described here. Evidence shows that melatonin restores mitochondrial crista morphology in the heart of leptin-deficient mice [73], but how melatonin counteracts metabolic alterations in skeletal muscle resulting from leptin deficiency/resistance has yet to be investigated. Our results demonstrate that melatonin optimizes OXPHOS efficiency by complex I and II in the muscle fibers of ob/ob mice. Nevertheless, melatonin could not modulate fatty acid β-oxidation but maintained increased proton leak respiration and reduced mitochondrial oxidative damage as in leptin-deficient mice, which suggests mitochondrial uncoupling for the suppression of ROS production. This is in sharp contrast to the downregulation of mtHK-II in melatonin-treated ob/ob mice, since mtHK-II promotes mitochondrial uncoupling to curtail ROS generation and maintain anaerobic respiration in the fed state [43]. This can be related to the recent finding that melatonin acts as a natural uncoupler to directly protect the mitochondria against oxidative stress [74]. This hypothesis of “uncoupling to survive” via melatonin action in ob/ob mice is supported by the restoration of mitochondrial biogenesis response and porin expression, suggesting mitochondrial mass recovery. Simultaneously, melatonin-enhanced *Ppargc1a* positively affects the expression of ROS-detoxifying enzymes [75], protecting muscle from leptin-deficiency-induced oxidative stress. We also showed that melatonin reduces mitochondrial cytochrome c and regulates apoptosis through increasing cytosolic Bax and re-establishing cyclophilin D, probably through its radical scavenging function. Interestingly, melatonin also reduced cytosolic oxidative damage and increased cytosolic antioxidant activities, exerting a protective effect against the damage induced by peroxisomal β-oxidation. Overall, our findings suggest that melatonin acts both directly and indirectly in protecting mitochondria and improving their efficiency while allowing for the partial restoration of glucose metabolism by favoring cytosolic HK-II. This is further reinforced by melatonin-induced *Pparg* enhancement, which is crucial for leptin secretion and glucose uptake upregulation, controlling energy homeostasis, and improving insulin sensitivity [76]. Melatonin was also found to modulate mitochondrial dynamics. In contrast to ob/ob mice, which showed MFN1-mediated mitochondrial fusion, melatonin-treated animals exhibited reduced MFN1 but presented high levels of MFN2. MFN2 mutation in trophoblast cells leads to the fragmentation of mitochondrial tubules, highlighting the functional importance of MFN2 for embryonic development in the context of a high metabolic rate [77]. Moreover, MFN2-mediated fusion re-establishment in mice with cachexia attenuates skeletal muscle loss [78]. Additionally, melatonin has been shown to increase MFN2 response and prevent myocardial infarction injury [79]. Altogether, these findings strongly support the therapeutic benefits of melatonin in targeting mitochondrial metabolism alterations in cases with leptin deficiency.

These bioenergetic actions of melatonin were accompanied by other metabolic-related effects that favor cell survival and maintenance. It is well known that mTOR/AMPK signaling is closely involved in the regulation of cellular homeostasis at multiple levels [80,81]. Interestingly, melatonin was found to ameliorate cellular damage through the activation of AMPK signaling [82]. Moreover, recent findings support the protective role of melatonin by promoting autophagy via the activation of the AMPK pathway [83]. It was reported that melatonin is a potent activator of autophagy via the mTOR/AMPK pathway, being even capable of counteracting the effects of inhibitors [84]. However, although melatonin treatment in ob/ob mice showed further mTOR inhibition, both autophagic flux and p62 were reduced, supporting the previously described protective role of melatonin against protein damage in the liver induced by leptin deficiency [85]. We found that melatonin transforms the exacerbated stress induced by obesity into mild oxidative stress, as indicated by the lower macroautophagy response and higher CMA, whose activation is mediated to counteract mild oxidative stress [86]. Consequently, this response contributes to reducing misfolded protein accumulation, which in turn leads to the downregulation of IRE1α/XBP1 and eIF2α pathways. However, the ATF6 pathway of the UPR was re-established by melatonin, suggesting a reduction in lipid anabolism. Then, melatonin seems to modulate the disrupted nutrient signaling induced by leptin deficiency and improve proteostasis by reducing the accumulation of protein aggregates and counteracting ER stress. Moreover, MFN2 has emerged as a linker of mitochondrial function, as ER stress and insulin signaling are essential for glucose homeostasis. MFN2 deficiency triggers hydrogen peroxide production, ER stress, and active JNK [87,88]. Then, the upward regulation of MFN2 induced by melatonin in ob/ob mice in this study further supports the finding that this hormone plays a crucial role in the modulation of all these pathways, thus improving skeletal muscle function.

Melatonin also exerts beneficial effects on the quality of skeletal muscle by modifying fuel utilization and targeting the switch between oxidative type I fibers to glycolytic type II fibers. Surprisingly, the most relevant finding is that melatonin restores mitochondrial ATP and reduces cytosolic ATP content in ob/ob animals, suggesting that this neurohormone is capable of balancing the rate of ATP generation with utilization.

Overall, our research is of paramount importance to better understand the effect of leptin on the regulation of cellular mechanisms that are critical for maintaining skeletal muscle quality. Here, we provided an overview of the cellular interactome of leptin-deficiency-induced obesity, but various limitations arose throughout the study that did not allow us to decipher the mechanistic role of leptin in some pathways. One of the limitations worth mentioning is the small size of the skeletal muscle samples extracted from the hind limbs. The implementation of certain state-of-the-art techniques, such as those used to characterize cellular bioenergetics, as well as mitochondrial and cytosolic isolation, requires a high sample quantity. Although these assays have contributed to uncovering various gaps in obesity research, new unknowns have emerged and must be examined in the future. Likewise, we used a murine model to test the effect of the lack of leptin on metabolism and muscle quality. However, it would be interesting to also test it in other murine or animal models in order to contrast the obtained results. Moreover, continuous research into the modulatory effect of melatonin on molecular mechanisms affected by leptin deficiency is needed to develop targeted therapies.

## 5. Conclusions

In the present work, leptin signaling was identified as a critical factor that is involved in the modulation of muscle metabolism and cell fate, especially by acting on mitochondrial function and quality control mechanisms. Here, we demonstrated that leptin deficiency induces metabolic reprogramming in skeletal muscle, which facilitates the acquisition of an energy conservation profile and compromises muscle mass and functional performance. We also found that melatonin has potential musculoskeletal benefits due to the pleiotropic effects of leptin mutation. Our study proved for the first time that melatonin regulates cellular metabolism and quality control mechanisms that actively recover muscle integrity. Therefore, melatonin could be a potential therapeutic agent for leptin-related disorders by partly overcoming the effects of leptin deficiency in several physiological functions and by regulating mitochondrial bioenergetics and cytosolic metabolism.

## Figures and Tables

**Table 1:** Gene accession numbers.

Gen		
*Acaca*	Mus musculus acetyl-Coenzyme A carboxylase alpha	XM_011248667
*Adipor1*	Mus musculus adiponectin receptor 1	NM_001306069
*Adipor2*	Mus musculus adiponectin receptor 2	NM_197985
*Atp2a1*	Mus musculus calcium-transporting ATPase	AY081946
*Calstabin1*	Mus musculus FK506 binding protein 1a	NM_008019
*Gapdh*	Mus musculus glyceraldehyde-3-phosphate dehydrogenase	NM_001289726
*Insr*	Mus musculus insulin receptor	NM_010568
*Jnk*	Mus musculus mitogen-activated protein kinase 8	NM_016700
*Lamp2a*	Mus musculus lysosomal-associated membrane protein 2	NM_001017959
*Ppargc1a*	Mus musculus peroxisome proliferator-activated receptor-gamma, coactivator 1 alpha	NM_008904
*Ppara*	Mus musculus peroxisome proliferator-activated receptor-alpha	XM_011245516
*Pparg*	Mus musculus peroxisome proliferator-activated receptor-gamma	NM_001127330
*Ryr1*	Mus musculus ryanodine receptor 1	XM_036152814
*Tfam*	Mus musculus transcription factor A, mitochondrial	NM_009360
*Xbp1*	Mus musculus X-box binding protein 1	NM_001271730

## Data Availability

No new data were created or analyzed in this study. Data sharing is not applicable to this article.

## Supplementary Materials

The following supporting information can be downloaded at: https://www.mdpi.com/article/10.3390/antiox12111962/s1, Figure S1: Macroscopic images of lower limb; Figure S2: Leptin deficiency triggers an inflammatory response; Figure S3: Melatonin remodels fatty acid metabolism in skeletal muscle from ob/ob mice. Table S1: Anthropometric indicators of leptin-deficiency-induced obesity.
