# Supplementary material for: Melatonin Alleviates the Impairment of Muscle Bioenergetics and Protein Quality Control Systems in Leptin-Deficiency-Induced Obesity
Source: Antioxidants (Basel). 2023 Nov 3;12(11):1962. doi: 10.3390/antiox12111962 (PMC10669624; doi:10.3390/antiox12111962)
Supplement: Supplementary file 1 [file antioxidants-12-01962-s001.zip › antioxidants-2640572-supplementary.pdf]

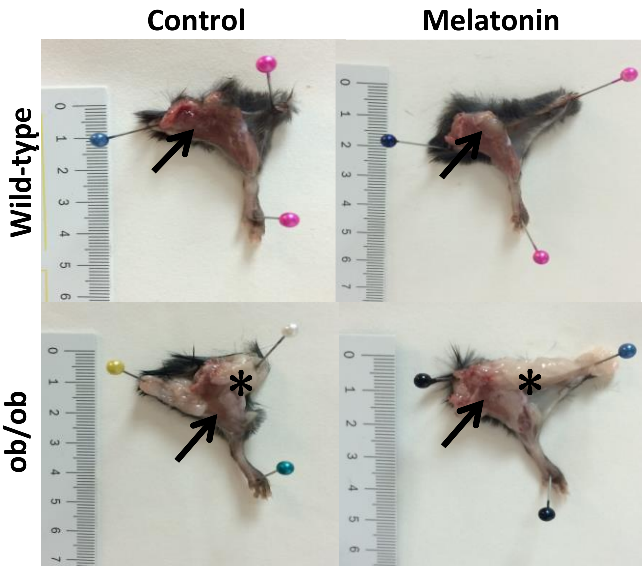

Figure S1. Macroscopic images of lower limb. Note the presence of a high amount of fat mass (asterisks) in ob/ob mice. Note that the color of skeletal muscle shifted from dark red in wild-type to pale red in ob/ob (arrows) which could be due to an increase in fat infiltration.

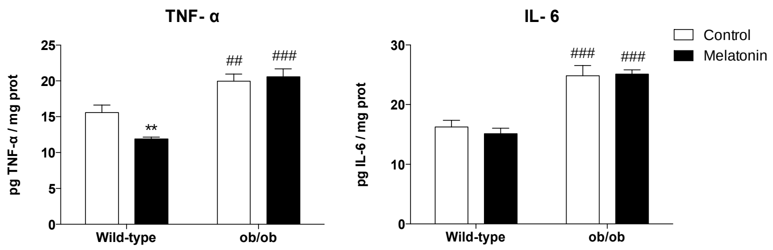

Figure S2. Leptin deficiency triggers an inflammatory response. Inflammation levels through the determination of TNF-α and IL-6 markers. Data are means ± SD. Statistical comparisons: # wild-type vs. ob/ob; * control vs. melatonin. The main effects of leptin deficiency and melatonin treatment were detected using a two-way ANOVA (n = 8). The number of symbols marks the level of statistical significance: one for $P < 0.05$, two for $P < 0.01$, and three for $P < 0.001$.

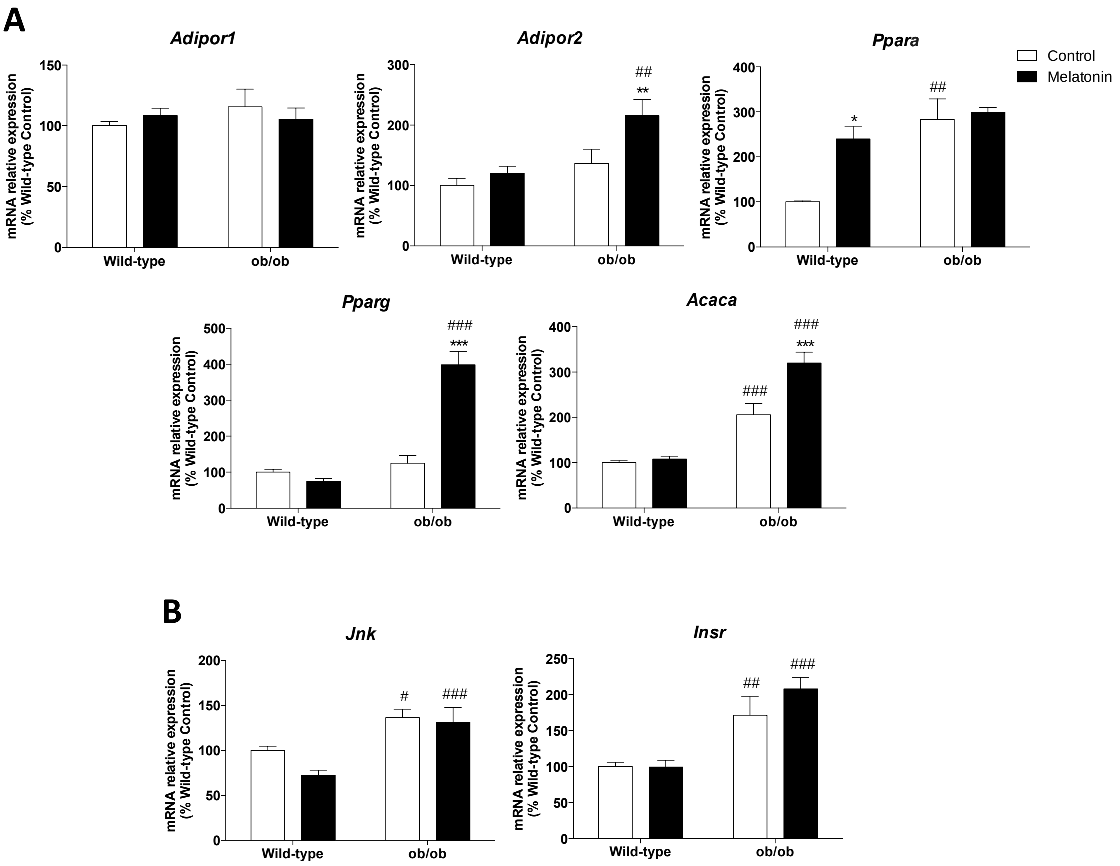

Figure S3. Melatonin remodels fatty acid metabolism in skeletal muscle from ob/ob mice. A, Relative mRNA expression of genes involved in β-oxidation (*Adipor1*, *Adipor2*, and *Ppara*) and in fatty acid synthesis (*Pparg* and *Acaca*) pathways. Data are means ± SD expressed as a percentage of wild-type control mice. B, Relative mRNA expression of genes implicated in insulin signaling (*Jnk* and *Insr*). Data are mean ± SD expressed as a percentage of wild-type control mice. Statistical comparisons: [#] wild-type vs. ob/ob; [*]Control vs. melatonin. The main effects of leptin deficiency and melatonin treatment were detected by two-way ANOVA (n = 8). The number of symbols marks the level of statistical significance: one for $P < 0.05$, two for $P < 0.01$ and three for $P < 0.001$.

Table S1. Anthropometric indicators of leptin-deficiency-induced obesity.

	Wild-type		ob/ob	
	**Control**	**Melatonin**	**Control**	**Melatonin**
**N**	8	8	8	8
**Food intake** (g/day)	3.27 ± 0.07	3.02 ± 0.22	4.82 ± 0.17 ###	5.17 ± 0.47 ###
**Body weight at basline** (g)	17.69 ± 1.37	17.69 ± 1.68	34.95 ± 2.93 ###	32.74 ± 2.67 ###
**Body weight at sacrifice** (g)	25.57 ± 0.94	23.98 ± 1.16	50.08 ± 3.82 ###	49.58 ± 2.59 ###
**Body weight changes** (g)	6.80 ± 1.54	6.30 ± 0.57	15.13 ± 1.85 ###	16.84 ± 2.01 ###
**Muscle weight at sacrifice** (g)	3.61 ± 0.36	3.66 ± 0.14	2.64 ± 0.07 ###	2.60 ± 0.19 ###
**Peripheral Fat weight at sacrifice** (g)	0.37 ± 0.06	0.38 ± 0.03	7.36 ± 0.54 ###	8.34 ± 1.10 ###
**Lower limb perimeter** (cm)	2.53 ± 0.24	2.68 ± 0.24	6.98 ± 0.67 ###	7.00 ± 0.66 ###
**Body Mass Index (BMI)** (kg/m^2)	3.53 ± 0.13	3.32 ± 0.16	6.18 ± 0.47 ###	6.21 ± 0.07 ###
**Skeletal Muscle Index (SMI)** (%)	13.08 ± 2.02	14.69 ± 1.46	4.98 ± 0.67 ###	5.11 ± 0.40 ###
**Fat Mass Index (FMI)** (g/m^2)	51.87 ± 8.66	53.15 ±3.62	948.15 ± 105.54 ###	1195.17 ± 231.32 ###
**Limb-Appendicular Skeletal Muscle Mass Index (L-ASMI)** (g/m^2)	461.82 ± 74.36	487.20 ± 49.06	308.64 ± 50.98 ##	320.10 ± 28.34 ###

Note: Data are means ± SD. Statistical comparisons: $^{\#}$ wild-type vs. ob/ob; $^{\#\#}P < 0.01$, $^{\#\#\#}P < 0.001$.
